# Heart Rate Variability and Peripheral Oxygen Saturation Levels in Chronic Obstructive Pulmonary Disease

**DOI:** 10.7759/cureus.77401

**Published:** 2025-01-13

**Authors:** Jeena P A, Bilal Mohideen, Abdul Rahman Ashraf, Swaliha M Thaha, Maheen Abu Shajahan, Ijaz Nazar, Rocky G Nair, Kannanunni KR, Nihala Thajudeen, Khadeeja Ashraf

**Affiliations:** 1 Department of Physiology, Sree Uthradom Thirunal (SUT) Academy of Medical Sciences, Thiruvananthapuram, IND; 2 Department of Medicine and Surgery, Al-Ameen Medical College, Vijayapura, IND; 3 Department of Emergency Medicine, Al Arif Hospital, Thiruvananthapuram, IND; 4 Department of Emergency Medicine, Ananthapuri Hospitals and Research Institute, Thiruvananthapuram, IND; 5 Department of Internal Medicine, Government Medical College, Pudukkottai, Pudukkottai, IND; 6 Department of Emergency Medicine, PRS Hospital, Thiruvananthapuram, IND; 7 Department of Internal Medicine, Annapoorana Medical College and Hospitals, Salem, IND; 8 Department of Internal Medicine, Danylo Halytsky Lviv National Medical University, Lviv, UKR; 9 Department of Emergency Medicine, Azeezia Medical College and Hospital, Thiruvananthapuram, IND; 10 Department of Internal Medicine, Government Sivagangai Medical College Hospital, Sivaganga, IND; 11 Department of General Practice, Dr. Safarulla's Polyclinic, Thiruvananthapuram, IND

**Keywords:** autonomic dysfunction, cardiopulmonary morbidity, chronic obstructive pulmonary disease, disease severity, heart rate variability, peripheral oxygen saturation

## Abstract

Chronic obstructive pulmonary disease (COPD) is a progressive respiratory disorder characterized by persistent airflow limitation, significantly impacting morbidity and mortality worldwide. The disease is often associated with autonomic nervous system (ANS) dysfunction, which can adversely affect cardiac function and overall prognosis. This study investigates the relationship between heart rate variability (HRV), a non-invasive marker of autonomic regulation, and peripheral oxygen saturation (SpO_2_) levels across different stages of COPD. The relevance of autonomic dysfunction in clinical practice is underscored by its potential to inform management strategies aimed at reducing cardiovascular risk and improving patient outcomes.

This prospective, hospital-based cross-sectional analysis included 80 patients aged 40-75 years, diagnosed with COPD based on spirometry criteria. Participants were grouped into four severity categories (Stages I-IV) according to the Global Initiative for Chronic Obstructive Lung Disease (GOLD) guidelines. HRV was assessed using five-minute recordings with a 16-channel polygraph machine, and SpO_2_ levels were measured via pulse oximetry. Both time-domain (standard deviation of normal-to-normal intervals (SDNN) and root mean square of successive differences (RMSSD)) and frequency-domain (low frequency (LF), high frequency (HF), and LF/HF ratio) HRV parameters were analyzed to evaluate autonomic function. Statistical analysis was performed using analysis of variance (ANOVA) to identify variations across the severity groups.

The findings revealed a statistically significant decline in HRV indices with increasing COPD severity. Specifically, the standard deviation of normal-to-normal intervals (SDNN) decreased from an average of 64.74 ms in Stage I to 37.12 ms in Stage IV, and the root mean square of successive differences (RMSSD) declined from 60.41 ms to 39.36 ms. Frequency-domain analysis showed an increase in the low-frequency/high-frequency (LF/HF) ratio, from 0.69 in Stage I to 4.72 in Stage IV, indicating a shift toward sympathetic dominance. Additionally, SpO_2_ levels decreased significantly from 98% in Stage I to 91.55% in Stage IV, reflecting the impact of hypoxemia on disease progression.

This study highlights the importance of early detection and monitoring of autonomic imbalance in COPD patients. HRV and SpO_2_ levels are identified as potential markers for disease severity, advocating for their integration into routine clinical evaluations. While significant associations were observed, the study's observational nature limits its ability to establish causation. Future research should focus on longitudinal assessments to better understand the prognostic value of HRV and SpO_2_ and guide targeted therapeutic interventions. Addressing autonomic and respiratory dysfunctions holistically could enhance the quality of life and reduce the burden of comorbidities in COPD patients. While our findings highlight the potential value of integrating autonomic function assessment in clinical evaluations, it is important to note that the study's conclusions are drawn from a relatively small sample size. Further large-scale studies are necessary to validate these results and assess their applicability in broader clinical practice.

## Introduction

According to the Global Initiative for Chronic Obstructive Lung Disease (GOLD) (2023) [1], chronic obstructive pulmonary disease (COPD) is defined as a "heterogeneous lung condition characterized by chronic respiratory symptoms (dyspnea, cough, expectoration, and/or exacerbations) due to abnormalities of the airways (bronchitis and bronchiolitis) and/or alveoli (emphysema) that cause persistent, often progressive, airflow obstruction". Chronic obstructive pulmonary disease (COPD) is a widespread respiratory disorder characterized by persistent respiratory symptoms and airflow limitation, resulting from airway and alveolar abnormalities typically caused by significant exposure to harmful particles or gases and is both largely preventable and treatable through pharmacological and non-pharmacological means [1-3].

COPD affects approximately 5% of the global population and stands as a leading cause of morbidity and mortality [4,5]. In India, it was the second highest cause of non-communicable disease deaths after heart disease in 2016, accounting for about 13% of all deaths and placing millions at risk [6]. The disease's progression often results in defects in gas exchange and reduced oxygen saturation levels, emphasizing the critical need for effective monitoring and management strategies.

Pulse oximetry has been highlighted as a valuable tool for assessing disease severity and guiding medical decisions in COPD patients [7]. Hypoxemia is a common complication, and supplemental oxygen therapy is recommended to prolong survival in affected individuals. However, it is crucial to maintain oxygen saturation levels below 92% to prevent potential oxidative stress damage that can negate the benefits of the therapy [8]. Maintaining oxygen saturation below 92% is particularly relevant for specific subgroups of COPD patients such as those with chronic hypercapnic respiratory failure, as it prevents oxygen-induced hypercapnia, a condition where excessive oxygen supplementation can worsen CO₂ retention to optimize oxygen therapy and minimize potential complications [8].

Cardiovascular complications are a significant concern in COPD, with cardiac failure being a common cause of mortality [1]. The increased afterload on the right ventricle due to elevated pulmonary vascular resistance, vascular damage, and hypoxic vasoconstriction can lead to the development of cor pulmonale and right heart failure [9,10]. Clinical manifestations of cor pulmonale in COPD may include elevated jugular venous pressure, hepatosplenomegaly, ascites, peripheral edema, and electrocardiographic changes such as sinus tachycardia, right axis deviation, right ventricular hypertrophy, and conduction abnormalities [11].

Beyond these cardiovascular issues, autonomic nervous system (ANS) dysfunction has been observed in COPD patients [12]. Heart rate variability (HRV) analysis is a non-invasive method to evaluate these autonomic alterations [12]. HRV refers to the variation in time intervals between consecutive heartbeats (R-R intervals) and reflects the dynamic interplay between sympathetic and parasympathetic influences on the sinoatrial node.

HRV can be assessed using time-domain and frequency-domain analyses. Time-domain methods examine the variability in beat-to-beat intervals over a specified time period and are reliable indicators of overall HRV and parasympathetic activity. Frequency-domain analysis, on the other hand, quantifies the distribution of low-frequency (LF) and high-frequency (HF) components within the heart rate signal. The low-frequency (LF) component represents both sympathetic and parasympathetic activity, while the high-frequency (HF) component is predominantly associated with parasympathetic modulation. The ratio of LF to HF (LF/HF ratio) is used to assess the sympathovagal balance, with higher values indicating sympathetic dominance. In COPD, autonomic dysfunction is common, with increased sympathetic and decreased parasympathetic activity, leading to reduced HRV. Time-domain metrics such as standard deviation of normal-to-normal intervals (SDNN) and root mean square of successive differences (RMSSD) reflect overall HRV and parasympathetic modulation, with lower values indicating impaired autonomic regulation in COPD. Frequency-domain metrics such as the LF/HF ratio assess the sympathovagal balance, where an increased ratio signals sympathetic dominance. These HRV changes in COPD are linked to worsened disease severity, increased cardiovascular risk, and poorer outcomes.

Patients with reduced HRV are at a greater risk of developing cardiovascular complications with studies consistently showing that individuals with COPD exhibit lower HRV compared to healthy counterparts of the same age group [13]. Studies have shown that COPD patients with lower HRV parameters are at an increased risk of cardiovascular events [13]. This association highlights the systemic impact of COPD on autonomic regulation and cardiovascular health. This reduction in HRV is associated with increased sympathetic tone at rest and disruptions in autonomic reflexes, contributing to a self-perpetuating cycle that exacerbates COPD pathophysiology and may influence mortality risk [13]. COPD appears to dampen excitatory pathways that regulate respiratory functions, cardiac autonomic control, and cardiovascular systems.

Given these associations, the present study aims to investigate cardiac autonomic dysfunction in patients with COPD. By examining the relationship between peripheral oxygen saturation levels and heart rate variability across different stages of disease severity, this research seeks to assess the risk of cardiovascular outcomes. The ultimate goal is to inform the development of novel therapeutic and preventive strategies to enhance the care and prognosis of patients suffering from COPD.

## Materials and methods

Study design

**Figure 1:**
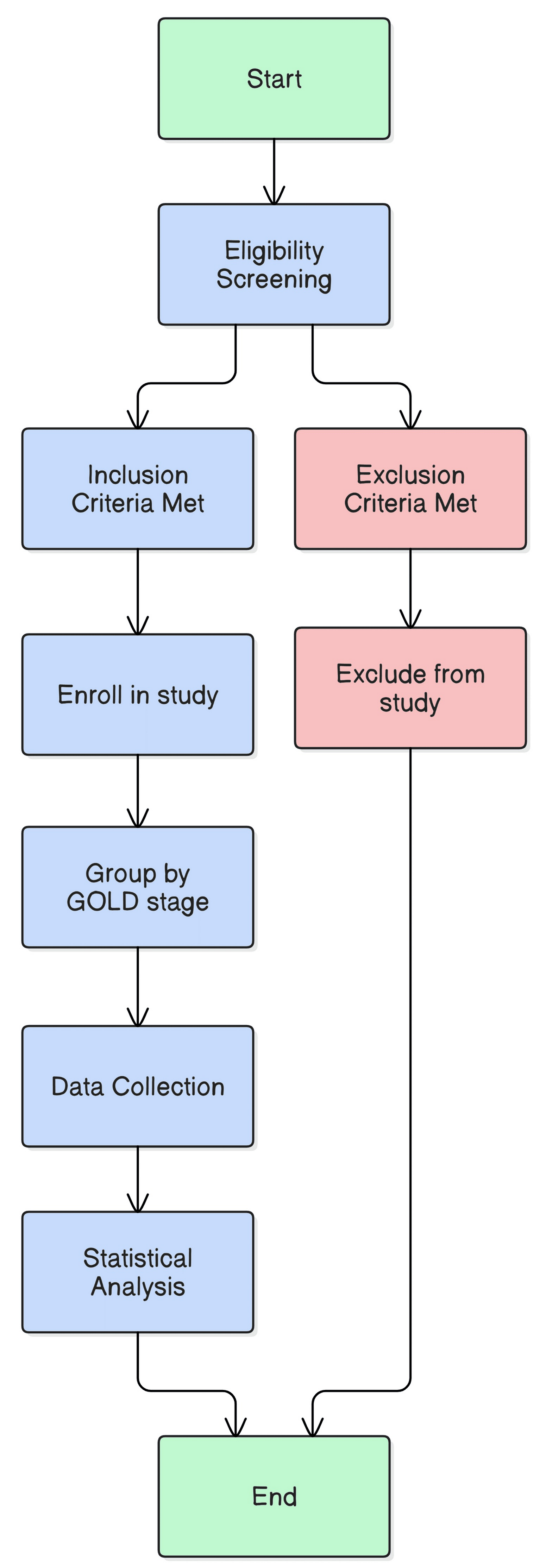
Study design Source: GOLD stage [1] GOLD: Global Initiative for Chronic Obstructive Lung Disease

This study is a prospective and hospital-based cross-sectional study as outlined in Figure 1.

The study utilizes a prospective, hospital-based cross-sectional design to examine the relationship between heart rate variability (HRV), SpO_2_ levels, and COPD stages. This design was chosen to allow for real-time data collection and minimize biases, ensuring that the findings are relevant to the current state of COPD patients. The study ensures a homogeneous sample by screening participants based on inclusion and exclusion criteria and grouping them by GOLD stage. Data is then collected on key physiological markers (HRV and SpO_2_), followed by statistical analysis to identify associations between these markers and disease severity. This approach aligns with the study's objective of understanding autonomic and cardiovascular dysfunction in COPD patients. The hospital-based setting ensures access to a controlled environment where accurate clinical data can be collected, including monitoring and diagnostic tools necessary for assessing HRV and SpO_2_. It also allows for close patient observation by healthcare professionals, ensuring proper management and timely data collection.

Study setting

This study was carried out at the Department of Pulmonology Medicine, Government T.D. Medical College, Alappuzha, Kerala. The region has a significant prevalence of respiratory diseases, including chronic obstructive pulmonary disease (COPD), making it an ideal location for studying this condition. Kerala has a high burden of smoking-related health issues, and the demographic characteristics of the population, such as age and lifestyle factors, may contribute to a greater number of COPD cases. The hospital is also well-equipped with the necessary diagnostic tools and expertise in pulmonology, enabling accurate data collection and management for the study.

Study period

This study was conducted over a period of 18 months from January 2020 to September 2021 after obtaining ethics committee approval. The 18-month duration from January 2020 to September 2021 was appropriate for the study objectives as it provided ample time to recruit a sufficient number of participants, ensuring a representative sample of COPD patients across different GOLD stages. This period allowed for comprehensive data collection, including baseline measurements of heart rate variability (HRV) and SpO_2_ levels, while also accounting for any variations in patient condition or seasonal factors that could affect these variables. Additionally, the extended timeline facilitated thorough follow-up, ensuring accurate data capture and reliable statistical analysis. The ethics committee approval process also ensures that the study was conducted with due consideration of patient safety and ethical guidelines. Overall, 18 months offered a balanced window for conducting rigorous research while maintaining patient care standards.

Study population

The study population included subjects aged between 40 and 75 years of both genders attending the outpatient Department of Pulmonology, Government T.D. Medical College, Alappuzha, who satisfied the inclusion and exclusion criteria (Table 1).

**Table 1:** Inclusion and exclusion criteria COPD: chronic obstructive pulmonary disease, ACE: angiotensin-converting enzyme

Inclusion criteria	Exclusion criteria
Patients aged between 40 and 75 years	Patients with acute exacerbation of COPD
COPD patients confirmed by spirometry	History of any known cardiac diseases except right heart failure
No change in medication in the last 2 weeks	Hypertension
Written consent to participate in the study	Diabetes
Stable COPD (no exacerbation in the last 4-6 weeks)	History of alcohol abuse or taking vasodilator drugs, ACE inhibitors, or antihypertensives
Willingness to comply with study procedures	Not willing to give consent

The exclusion criteria, which include patients with acute exacerbation of COPD, a history of cardiac diseases (except right heart failure), hypertension, diabetes, alcohol abuse, use of vasodilator drugs, angiotensin-converting enzyme (ACE) inhibitors, or antihypertensives, and those not willing to give consent, are designed to minimize confounding factors that could influence the study's findings. These conditions, particularly cardiac diseases and comorbidities such as hypertension and diabetes, can affect autonomic function, heart rate variability (HRV), and oxygen saturation (SpO_2_) levels. Including patients with such conditions might obscure the specific relationship between COPD and these physiological markers. By excluding patients with acute exacerbations, the study focuses on stable COPD cases, ensuring that the observed associations between HRV and SpO_2_ reflect the disease's chronic state rather than temporary exacerbations. Additionally, excluding individuals with alcohol abuse or on specific medications (such as ACE inhibitors or vasodilators) ensures that drug interactions or lifestyle factors do not interfere with the study's outcomes. However, these exclusions could limit the study's external validity, as the findings may not be generalizable to the broader COPD population, particularly those with comorbidities or other complicating factors commonly seen in clinical practice.

Sample size

A total of 80 patients with clinical and investigational features suggestive of chronic obstructive pulmonary disease (COPD) were studied. The sample size was calculated according to the study by Gupta et al. [14]. In their study, SpO_2_ means were 95.50 ± 1.41, 95.05 ± 2.42, 94.37 ± 2.28, and 93.05 ± 1.39 in Stages I, II, III, and IV, respectively. The following formula was used:

$n = \frac{Z^2_{1-\alpha/2} \cdot 2S_p^2}{d^2}$

where $S_p^2 = \frac{S_1^2 + S_2^2}{2}$.

Here, $S_1^2 = 2.28^2, S_2^2 = 1.39^2,d = 1.2, and \alpha = 95\%$.

Substituting the values, $S_p^2 = \frac{(2.28)^2 + (1.39)^2}{2}$ $S_p^2 = \frac{5.1984 + 1.9321}{2} = 3.56525$

Now, calculating $n$, $n = \frac{(1.96)^2 \cdot 2 \cdot 3.56525}{(1.2)^2}$ $n = \frac{3.8416 \cdot 7.1305}{1.44}$ $n = \frac{27.3944}{1.44} = 19.027$.

Rounding up, $n = 20 \, \text{per group}$.

For four groups (GOLD 1 to GOLD 4), the total sample size is $n_{\text{total}} = 20 \times 4 = 80$.

Sampling method

Participants fulfilling the inclusion criteria were selected, and those fulfilling the exclusion criteria were rejected. This study employed a convenience sampling method, where participants were selected based on their availability and willingness to participate at the hospital during the study period. While this approach facilitated efficient data collection, it may introduce selection bias, as the sample may not fully represent the broader COPD population. Patients with comorbidities or those not seeking treatment at the study hospital were excluded, which could limit the generalizability of the findings.

Study variables

The study variables were as follows: age, gender, body mass index (BMI), forced expiratory volume in one second (FEV1%), SpO_2_, and heart rate variability (HRV) (time- and frequency-domain analysis). Age and gender were included due to their influence on autonomic function and disease progression. BMI was considered as it impacts lung function and cardiovascular health, which could confound HRV and SpO_2_ relationships. FEV1% is crucial for assessing COPD severity, while SpO_2_ provides insights into oxygenation status, a key factor in COPD outcomes. HRV, assessed through time- and frequency-domain analyses, reflects autonomic dysfunction in COPD. Confounding factors such as age, gender, and BMI were controlled through statistical methods, and comorbidities or medications were excluded to isolate the effects of COPD on HRV and SpO_2_.

Time-domain indices

The time-domain indices included the mean R-R interval, the standard deviation for normal R-R intervals (SDNN), and the root mean square of successive differences between R-R intervals (RMSSD).

Frequency-domain measures

Frequency-domain measures included low-frequency (LF) normalized units, high-frequency (HF) normalized units, and the LF/HF ratio.

Data collection tools

The proforma is given in the Appendices for reference. For this study, a 16-channel polygraph machine, HRV analysis software (version 1.1), spirometer, and pulse oximeter were utilized based on their technical specifications, accuracy, and suitability for the research objectives. The polygraph machine was chosen for its ability to record multiple physiological signals simultaneously, essential for heart rate variability (HRV) analysis. The HRV analysis software was selected for its reliability in performing time- and frequency-domain analyses of HRV, a key measure of autonomic function. The spirometer was used to assess pulmonary function, specifically FEV1%, which is critical for COPD severity classification. The pulse oximeter provided a non-invasive measurement of oxygen saturation (SpO_2_), an important marker of oxygenation in COPD patients. While these instruments have not been specifically validated in COPD populations, they are widely used and trusted in clinical and research settings

Data collection procedure

Institutional ethics and research committee approval was obtained. Eligible patients were briefed about the study, and written informed consent was obtained. Participants were grouped into four stages of COPD based on the Global Initiative for Chronic Obstructive Lung Disease (GOLD) guidelines (Table 2) [1].

**Table 2:** GOLD guidelines Source: [1] GOLD: Global Initiative for Chronic Obstructive Lung Disease, FEV1: forced expiratory volume in one second

Stage	Severity	FEV1 (% predicted)
GOLD 1	Mild	FEV1 ≥ 80%
GOLD 2	Moderate	50% ≤ FEV1 < 80%
GOLD 3	Severe	30% ≤ FEV1 < 50%
GOLD 4	Very severe	FEV1 < 30%

**Figure 2:**
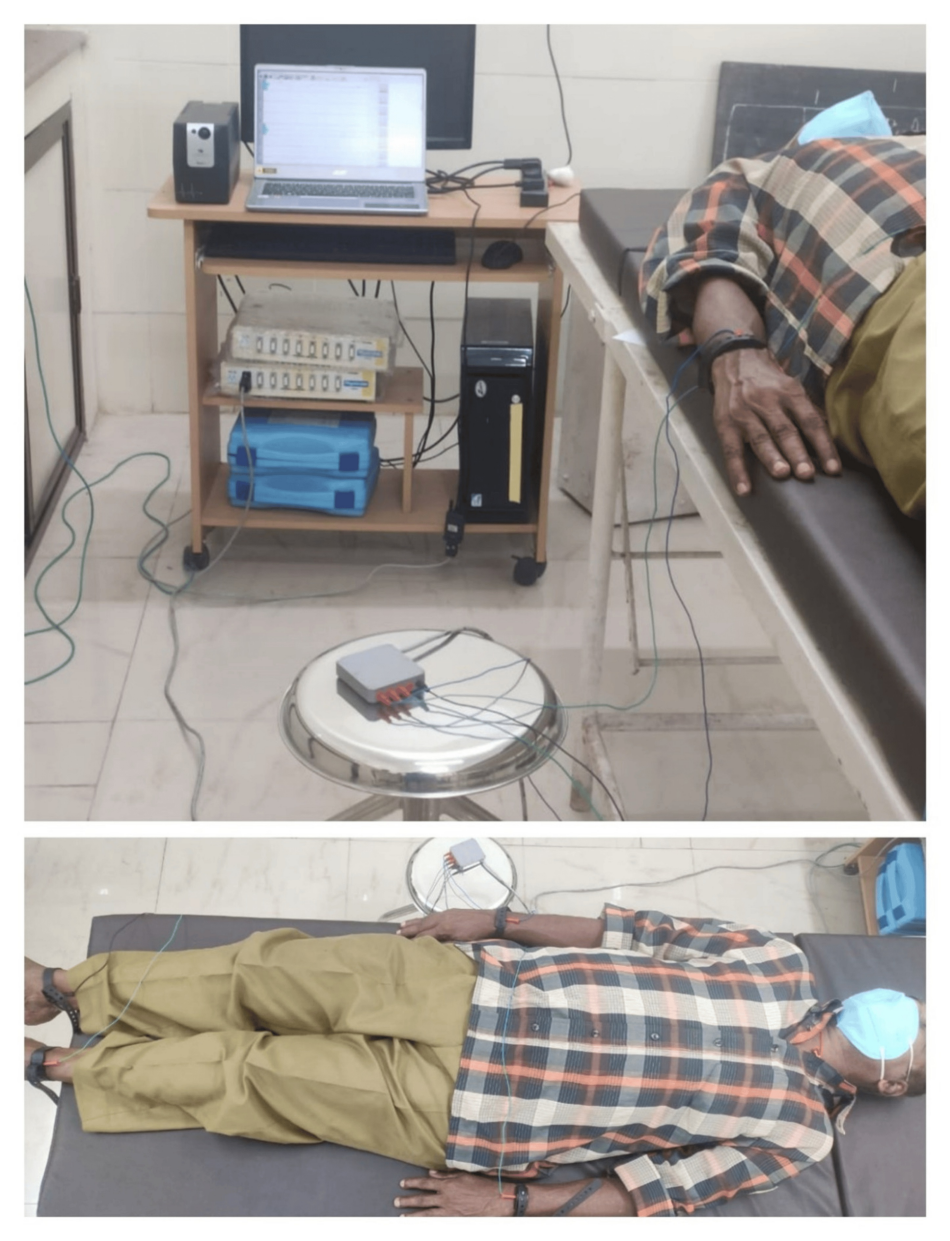
HRV recording in a subject HRV: heart rate variability

Assessment was conducted between 8 AM and 11 AM for all participants to avoid the influence of circadian rhythm. Participants were advised to leave their mobile phones, jewelry, watches, and coins with their attendants to avoid interference. Each subject was made to relax for 10 minutes in the supine position before data collection began. Electrocardiography (ECG) electrodes were attached to the participants and connected to the polygraph machine. The positive electrode was connected to the left arm, the negative electrode to the right arm, the reference electrode to the left foot, and the ground electrode to the right foot, using a four-lead ECG cable. Lead II ECG was recorded for five minutes in a quiet, acclimatized room. Participants underwent assessments for height, weight, and BMI. ECG and multiple SpO_2_ readings were averaged to minimize variability caused by motion artifacts or peripheral perfusion issues. HRV analysis was performed using a computerized 16-channel polygraph and HRV analysis software version 1.1 (Figure 2).

**Figure 3:**
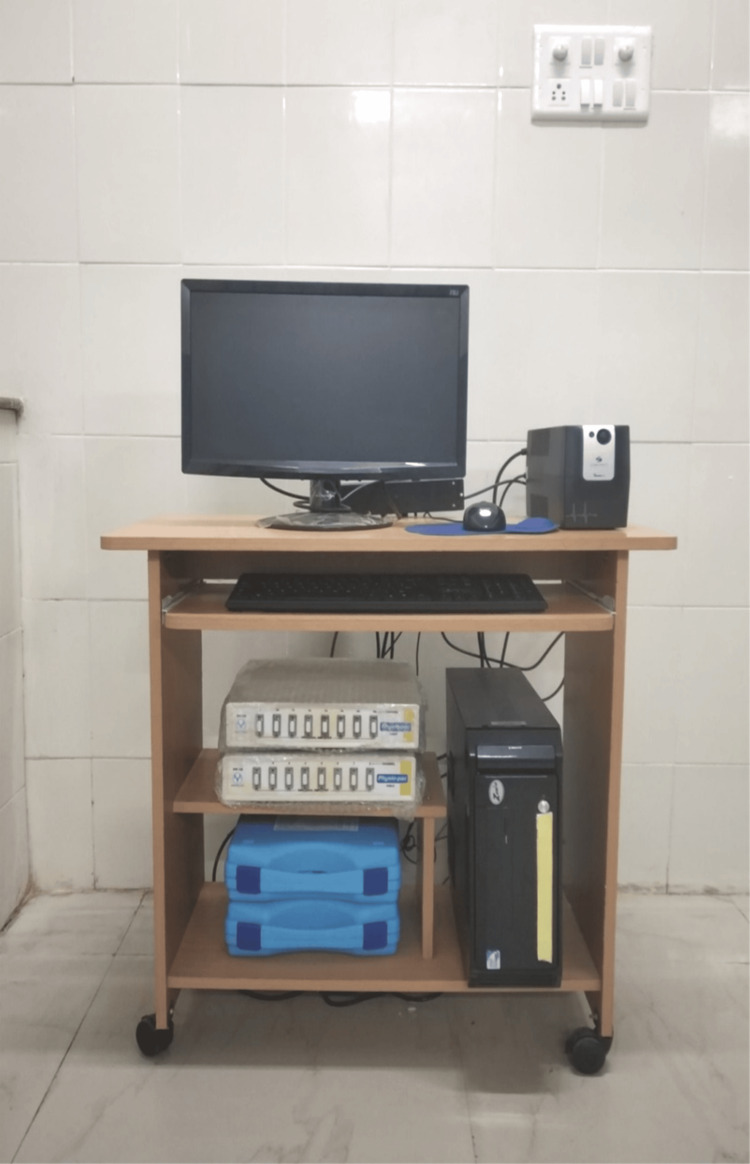
16-channel computer-based polygraph machine

This 16-channel computer-based polygraph machine, supplied by Medicaid Systems (Chandigarh, India), recorded physiological signals such as ECG, electromyography (EMG), and electroencephalography (EEG) (Figure 3).

Filters and software ensured real-time data storage and HRV analysis. The in-built HRV analysis software detected all R waves and computed R-R intervals from ECG recordings. Parameters included time-domain measures such as the mean R-R interval, SDNN, RMSSD, and frequency-domain measures such as LF, HF, and LF/HF ratio. The ECG signal was digitized and stored on a computer with the analysis software. The data gathered were analyzed for HRV, including time- and frequency-domain parameters. The final reports were displayed on A4 sheets along with their interpretations (Table 3).

**Table 3:** Final data COPD stage: chronic obstructive pulmonary disease stage, F: female, M: male, HT: height (cm), WT: weight (kg), BMI: body mass index (kg/m²), FEV1%: forced expiratory volume in one second (percentage of predicted), Mean RR: mean R-R interval (ms), SDNN: standard deviation of normal-to-normal RR intervals (ms), RMSSD: root mean square of successive differences (ms), LF (nu): low-frequency power (normalized units), HF (nu): high-frequency power (normalized units), LF/HF ratio: ratio of low-frequency to high-frequency power, SpO_2_: peripheral oxygen saturation (%)

SI number	COPD stage	Gender	Age	HT	WT	BMI	FEV1%	Mean R-R	SDNN	RMSSD	LF (nu)	HF (nu)	LF/HF ratio	SpO_2_
1	Stage I	F	47	164	62	23.05	93	811	74.4	64.5	26.548	73.482	0.361	98
2	Stage I	M	51	156	65	25.39	92	828	75.4	73	28.349	71.651	0.396	99
3	Stage I	M	63	165	69	25.34	91	851	74.6	66	29.54	70.46	0.419	98
4	Stage I	M	46	165	85	31.22	90	933	64.9	60.9	30.668	69.332	0.442	98
5	Stage I	M	48	174	68	22.46	90	798	58.4	62.9	33.296	66.704	0.499	97
6	Stage I	M	57	159	47	18.59	89	815	76.6	63.14	36.233	63.767	0.568	99
7	Stage I	F	62	165	60	22.03	89	819	55.5	59.3	36.233	63.767	0.568	99
8	Stage I	M	56	164	56	20.82	88	993.9	84.6	58.9	33.432	66.568	0.502	98
9	Stage I	M	57	172	62	20.95	87	775	55.7	56.4	37.418	62.582	0.598	98
10	Stage I	M	65	167	66	23.66	87	790	61.1	62.9	37.422	62.58	0.598	99
11	Stage I	M	60	155	58	24.14	87	802	58.7	61.8	43.308	56.692	0.764	96
12	Stage I	F	64	165	58	21.3	86	800	62.1	54.4	40.57	46.19	0.878	97
13	Stage I	M	59	162	64	24.38	86	816	67.9	56	48.885	51.115	0.956	98
14	Stage I	M	51	158	62	24.83	86	773	50.4	59.7	47.23	52.77	0.895	97
15	Stage I	M	66	160	58	22.65	85	757	65.1	57.9	48.88	51.12	0.956	99
16	Stage I	M	53	164	65	24.16	85	769	66.2	58.1	49.995	51	0.98	97
17	Stage I	M	57	158	62	24.83	84	781	63.1	57.2	49.975	50.025	0.999	98
18	Stage I	F	48	164	63	23.42	83	763	58.7	68.4	51.54	50.005	1.031	98
19	Stage I	M	63	160	55	21.48	82	772	61.2	54.1	33.42	66.7	0.501	99
20	Stage I	M	57	158	60	24.03	81	736	60.2	52.7	40.57	46.19	0.878	98
21	Stage II	M	63	165	69	25.34	79	728	78.6	66.9	53.481	46.519	1.15	95
22	Stage II	M	51	158	62	24.83	78	889.7	69.7	73.9	53.854	46.5	1.158	96
23	Stage II	M	70	160	65	25.39	77	845.3	70.4	72.3	53.525	46.476	1.152	97
24	Stage II	M	63	160	48	18.75	75	840	75.4	61.2	54.225	45.749	1.185	94
25	Stage II	M	65	160	68	26.56	75	851	79	65.7	54.335	45.8	1.186	95
26	Stage II	M	62	163	55	20.7	69	868	64.5	55.1	55.192	44.808	1.232	94
27	Stage II	M	60	160	48	18.75	69	936	58.6	55.5	53.525	40.806	1.312	95
28	Stage II	M	58	162	50	22.03	68	750	49.3	41.3	56.248	44.011	1.278	94
29	Stage II	M	54	152	64	27.7	68	761	40.7	31.5	57.453	43.998	1.306	95
30	Stage II	F	52	162	57	21.71	67	780	42.3	43.5	59.478	42.89	1.387	96
31	Stage II	M	57	160	56	21.87	67	721	40.18	31.4	59.4	42.8	1.388	96
32	Stage II	M	53	162	68	25.91	67	800.9	55.1	55.4	60.457	41.577	1.454	97
33	Stage II	M	60	160	46	17.96	66	721	42.7	42.8	60.788	40.677	1.494	96
34	Stage II	M	56	157	64	25.96	66	875	47.4	37	62.344	37.657	1.656	97
35	Stage II	M	72	157	56	22.71	65	774	44.1	38	62	37.1	1.671	97
36	Stage II	F	52	149	48	21.62	65	748	40.1	35.8	64.797	35.869	1.807	98
37	Stage II	M	63	163	55	20.7	64	750	48.2	33.8	66.21	33.803	1.959	97
38	Stage II	M	48	158	62	24.83	60	698	49.18	41.16	65.688	34.898	1.882	98
39	Stage II	M	62	152	45	19.47	57	727	45.7	40.8	65.099	34.327	1.896	95
40	Stage II	M	65	162	57	21.71	57	725.3	40.6	42.7	67.898	34.987	1.941	96
41	Stage III	M	65	168	60	21.3	49	721	45.9	40.9	67.509	32.491	2.078	94
42	Stage III	M	70	166	51	18.5	49	745	45.3	45.2	67.65	32.2	2.101	92
43	Stage III	M	67	162	45	17.1	49	615	33	43.9	67.657	32.343	2.092	95
44	Stage III	M	50	165	51	18.7	48	638	39.9	41.5	68.387	31.613	2.163	94
45	Stage III	M	77	167	67	24	48	728	39.7	45	68.451	31.549	2.17	93
46	Stage III	M	80	168	66	23.4	47	645	39.5	42.9	68.5	31.005	2.209	95
47	Stage III	M	67	168	55	21	46	590	38.7	39.4	68.872	31.128	2.213	94
48	Stage III	M	69	170	62	21.5	46	598	48	39.5	69.473	30.527	2.276	93
49	Stage III	M	60	162	56	21.3	45	610	49.9	43.5	70.654	29.676	2.381	94
50	Stage III	M	64	160	53	20.7	44	600	48.9	37.5	70.248	29.226	2.404	94
51	Stage III	M	65	165	49	18	44	623	42.8	39.9	71.304	28.696	2.485	95
52	Stage III	M	79	164	55	20.4	44	645	49.5	39.5	71.1	28.299	2.512	95
53	Stage III	M	50	167	52	18.6	43	733	32.5	39.4	71.668	28.787	2.49	94
54	Stage III	M	68	168	55	19.5	41	698	33	42.4	72.848	27.152	2.683	94
55	Stage III	M	66	167	60	21.5	40	622	37.4	41.9	72.848	27.152	2.683	94
56	Stage III	M	76	158	60	24	39	673	39.4	42	75.875	25.766	2.945	93
57	Stage III	F	72	156	43	17.7	37	612	33	53.9	78.875	26.879	2.935	95
58	Stage III	F	62	153	64	27.3	37	655	40	43.6	79.877	23.878	3.345	95
59	Stage III	M	66	160	50	19.5	36	589	40.9	41.1	79.2	22.898	3.459	94
60	Stage III	F	65	154	42	17.7	35	600	36	39	79.1	22	3.595	93
61	Stage IV	M	70	168	60	21.3	29	621	40	42.1	79.473	20.528	3.871	89
62	Stage IV	M	68	165	85	31.22	29	623	35.2	39.9	79.093	20.904	3.784	90
63	Stage IV	M	65	165	69	25.34	28	659	33	39.5	79.608	20.392	3.904	91
64	Stage IV	M	85	174	68	22.46	29	683	35.9	39.8	84.811	15.189	5.584	92
65	Stage IV	M	76	160	58	22.65	29	500	40.2	43.8	87.669	12.331	7.11	92
66	Stage IV	M	84	160	65	25.39	29	694.1	38.1	51	87.669	12.331	7.11	92
67	Stage IV	M	89	160	55	21.48	29	688.1	33.4	32.9	82.537	17.463	4.726	91
68	Stage IV	M	67	158	60	24.03	29	689	37.9	33	90.685	9.315	9.736	92
69	Stage IV	F	75	156	59	24.24	29	697.5	36.8	38.9	90.685	9.315	9.736	93
70	Stage IV	M	76	165	57	20.93	29	697.1	39.5	38.9	85.878	14.988	5.73	93
71	Stage IV	M	78	164	88	32.71862	29	654	43	37.5	86.988	13.875	6.269	92
72	Stage IV	M	86	170	80	27.68166	29	621.9	40.2	39.2	79.666	21.898	3.638	92
73	Stage IV	M	85	165	85	31.2213	29	599.6	36.1	38.5	77.876	20.757	3.752	93
74	Stage IV	M	85	158	83	33.24788	29	598.2	36.5	39	75.787	20.875	3.63	93
75	Stage IV	M	85	164	75	27.88519	29	627.9	34.6	39.1	67.786	32.868	2.062	91
76	Stage IV	M	50	169	80	28.01022	29	597	36.4	35	75.877	23.777	3.191	89
77	Stage IV	M	84	165	88	32.32323	29	678	34.6	41	78.987	20.788	3.8	90
78	Stage IV	M	79	164	56	20.82094	29	640	38.2	40.2	68.877	30.788	2.237	94
79	Stage IV	M	78	166	79	28.66889	29	687	34.7	39.9	68.868	31.877	2.16	92
80	Stage IV	M	79	160	80	31.25	29	734	38.1	38	69.877	30.767	2.271	90

The GOLD stages for each participant were assessed during the study based on spirometry tests conducted at the time of data collection, ensuring consistency and accuracy in categorizing disease severity according to the current clinical condition. The 8-11 AM time window was chosen to minimize circadian rhythm influence on heart rate variability (HRV) and peripheral oxygen saturation (SpO_2_) measurements, as this period represents a stable phase in the circadian cycle. Participants were acclimatized in a quiet, acclimatized room with a controlled temperature of 22-24°C, and noise levels were minimized to create a calm environment. They rested in the supine position for 10 minutes before recordings to stabilize heart rate and respiration. Proper electrode placement was ensured by cleaning the skin with alcohol swabs to reduce impedance, following standard ECG protocols for electrode positioning. HRV parameters, including SDNN, RMSSD, LF, and HF, were selected for their ability to provide comprehensive insights into autonomic function, with SDNN and RMSSD reflecting overall HRV and parasympathetic activity, and LF and HF assessing sympathovagal balance. The HRV analysis was conducted using version 1.1 of the software, validated for its reliability. Potential artifacts and outliers in ECG data were managed using built-in software filters and manual reviews, ensuring data integrity and accuracy.

Statistical analysis

Data were analyzed using Microsoft Excel (Microsoft Corp., Redmond, WA) and SAS (version 9.2) software. Categorical variables such as age group and sex were expressed as percentages and frequencies, while continuous variables such as BMI, FEV1, and SpO_2_ were expressed as mean ± standard deviation (SD). Univariate analysis, Chi-square tests, and one-way analysis of variance (ANOVA) tests determined significance, with P < 0.05 considered significant, which is a commonly accepted threshold in hypothesis testing in most scientific studies, indicating that the probability of the observed results occurring by chance is less than 5%.

Ethical considerations

The study was conducted following ethical guidelines, with approval from the institutional ethics committee. Written informed consent was obtained from all participants after being provided with a participant information sheet detailing the study.

Participants were thoroughly briefed about the study's purpose, procedures, and any potential risks or benefits during the consent process. They were informed that participation posed a minimal risk, primarily involving non-invasive measurements such as ECG and pulse oximetry. The benefits included contributing to research that may improve the understanding and management of COPD. Written informed consent was obtained after participants reviewed a detailed information sheet explaining the study.

To ensure data confidentiality, all collected information was anonymized and securely stored. Participants' personal identifiers were removed, and data was accessed only by authorized personnel. The study adhered to ethical guidelines as outlined by the Declaration of Helsinki and the Indian Council of Medical Research (ICMR) guidelines for biomedical research involving human participants.

Expected outcome of the study

A decrease in heart rate variability and peripheral oxygen saturation levels among COPD patients was expected, with greater decreases in the advanced stages of COPD. These expectations are based on prior studies demonstrating that the progressive impact of COPD on autonomic function and gas exchange efficiency, with reduced HRV and SpO_2_ levels, is closely associated with heightened cardiovascular risk due to exacerbated sympathetic overactivity and increased likelihood of adverse cardiovascular events [13].

## Results

Baseline characteristics

A total of 80 COPD patients participated in the study conducted between January 2020 and September 2021 at the Department of Pulmonology, Government T.D. Medical College, Alappuzha, Kerala. Participants were classified into four stages of COPD (Stage I-IV) based on GOLD guidelines [1]. The study period, spanning January 2020 to September 2021, allowed for data collection across multiple seasons, capturing potential environmental influences such as air pollution, humidity, and temperature fluctuations, which are known to impact COPD outcomes. Seasonal variations, including Kerala's monsoons and higher humidity, may have influenced respiratory health, contributing to symptom exacerbations or variations in oxygen saturation levels. Additionally, the timeline included the early phase of the COVID-19 pandemic, which could have affected participants' healthcare access and disease management. All participants had pre-existing COPD diagnoses confirmed via spirometry prior to recruitment as well as during data collection for this study, ensuring the study focused on stable cases without confounding factors from new diagnoses or recent exacerbations.

*Age Group Distributio*n

A significant association was observed between age groups and COPD severity (P < 0.001) (Table 4).

**Table 4:** Age group distribution Values are presented as numbers (% of cases), P-values by Chi-square test. P<0.05 is considered to be statistically significant. COPD: chronic obstructive pulmonary disease

COPD stage	41-50 years (number)	41-50 years (%)	51-60 years (number)	51-60 years (%)	61-70 years (number)	61-70 years (%)	≥71 years (number)	≥71 years (%)
Stage I	4	50.0	10	45.45	6	14.63	0	0.0
Stage II	1	12.5	10	45.45	8	19.51	1	11.11
Stage III	2	25.0	1	4.55	16	39.02	1	11.11
Stage IV	1	12.5	1	4.55	11	26.83	7	77.78
Total	8	100.0	22	100.0	41	100.0	9	100.0

Most participants were aged 61-70 years (50%). The majority of Stage III and IV patients were in the 61-70 and ≥71 age groups, whereas Stages I and II predominantly included younger participants. The age group distribution shows that younger individuals are more likely to have less severe COPD (Stages I and II), while older individuals predominantly have advanced stages (Stages III and IV). This trend suggests that age contributes to disease progression, likely due to prolonged exposure to risk factors and age-related declines in pulmonary function. These findings emphasize the need for early detection and intervention in younger patients to prevent progression, while older individuals may require comprehensive management to address advanced disease and associated comorbidities.

Mean Age Distribution

The overall mean age was 62.21 years, with a significant increase observed with disease severity (P < 0.001) (Table 5).

**Table 5:** Mean age distribution P-value by ANOVA. P < 0.05 is statistically significant. COPD: chronic obstructive pulmonary disease, ANOVA: analysis of variance, SD: standard deviation

COPD stage	Age range (years)	Mean ± SD (years)
Stage I	46-66	56.5 ± 6.37
Stage II	48-72	59.3 ± 6.43
Stage III	50-72	64.85 ± 5.79
Stage IV	50-75	68.2 ± 5.91

A progressive increase in mean age across COPD stages reflects an association between advancing age and COPD severity. Male participants constituted the majority (87.5%) across all stages in terms of gender distribution. No significant association was found between gender and COPD severity (P = 0.51). The progressive increase in mean age across COPD stages aligns with existing literature, reflecting the cumulative impact of risk factors such as smoking and environmental exposures over time. However, the overlap in age ranges across stages highlights the heterogeneity in disease progression due to individual factors such as genetic predisposition, environmental exposure, and comorbidities, as well as potential delays in diagnosis. The male predominance (87.5%) likely reflects historical smoking patterns but may also indicate sampling bias, influenced by socio-cultural factors or limited access to healthcare among women. The absence of a significant association between gender and COPD severity (P = 0.51) could stem from the underrepresentation of female participants, reducing the statistical power to detect differences. This gender imbalance limits the generalizability of the findings, as women with COPD may exhibit distinct symptom profiles and disease trajectories. Future studies should aim for balanced gender representation to ensure broader applicability of the results.

Mean Height and Weight Distribution

The mean height and weight of the participants across COPD stages are presented (Table 6 and Table 7).

**Table 6:** Height distribution P-value by ANOVA. P < 0.05 is statistically significant. COPD: chronic obstructive pulmonary disease, ANOVA: analysis of variance, SD: standard deviation

COPD stage	Height range (cm)	Mean ± SD (cm)
Stage I	155-174	162.75 ± 4.92
Stage II	149-165	159.1 ± 4.09
Stage III	153-170	163.4 ± 5.06
Stage IV	156-174	163.8 ± 4.5

**Table 7:** Weight distribution P-value by ANOVA. P < 0.05 is statistically significant. COPD: chronic obstructive pulmonary disease, ANOVA: analysis of variance, SD: standard deviation

COPD stage	Weight range (kg)	Mean ± SD (kg)
Stage I	47-85	62.25 ± 7.32
Stage II	45-69	57.15 ± 7.83
Stage III	42-67	54.8 ± 7.22
Stage IV	55-88	71.5 ± 11.95

COPD severity showed a significant correlation with age, height, and weight. The majority of Stage III and IV patients were older, with reduced weight reflecting disease progression. The ranges of height in each COPD stage overlap significantly, which may limit the ability to draw definitive conclusions about height as a distinguishing factor across stages. This overlap suggests that height alone may not be a reliable indicator of COPD severity. However, the observed significant correlation between weight and COPD severity aligns with the known pathophysiology of the disease. Weight tends to decrease as COPD progresses, particularly in advanced stages, where cachexia, a syndrome characterized by weight loss, muscle wasting, and decreased body fat, is common. This is often attributed to the increased metabolic demands of chronic respiratory effort, systemic inflammation, and reduced appetite. The fluctuations in weight observed across different stages in this study reflect these expected outcomes, with lower weight in advanced stages supporting the link between COPD severity and the systemic effects of chronic illness.

Body Mass Index (BMI)

BMI distribution among COPD stages is provided in Table 8.

**Table 8:** Body mass index distribution P-value by ANOVA. P < 0.05 is statistically significant. COPD: chronic obstructive pulmonary disease, BMI: body mass index, ANOVA: analysis of variance, SD: standard deviation

COPD stage	BMI range (kg/m²)	Mean ± SD (kg/m²)
Stage I	18.59-31.22	23.44 ± 2.54
Stage II	17.96-27.7	22.73 ± 2.91
Stage III	17.1-27.3	20.59 ± 2.6
Stage IV	20.82-33.24	26.64 ± 4.33

It was observed that Stage IV patients had a higher mean BMI, which seems to contradict the expected trend of weight loss in severe COPD due to cachexia. Possible explanations for this include fluid retention, often associated with right heart failure and cor pulmonale, which can occur in advanced COPD, or other confounding factors such as variations in muscle mass or the use of medications that may impact weight. Additionally, the BMI ranges overlap significantly between the different stages, similar to the observed overlap in height and weight distributions. This overlap may limit the ability to clearly differentiate between stages based solely on BMI. It should be interpreted cautiously within the broader context of clinical assessment.

Forced Expiratory Volume in the First Second (FEV1%)

FEV1% distribution, an important marker of lung function, is presented in the following table. A significant decline in FEV1% was observed across COPD stages (P < 0.001), indicating worsening pulmonary obstruction (Table 9).

**Table 9:** Forced expiratory volume in the first second distribution P-value by ANOVA. P < 0.05 is statistically significant. FEV1%: forced expiratory volume in the first second, COPD: chronic obstructive pulmonary disease, ANOVA: analysis of variance, SD: standard deviation

COPD stage	FEV1% range (%)	Mean ± SD (%)
Stage I	81%-93%	87.05 ± 3.25
Stage II	57%-79%	67.95 ± 6.3
Stage III	35%-49%	43.35 ± 4.65
Stage IV	28%-29%	28.95 ± 0.22

FEV1% showed a marked decline with advancing stages (28%-29%), reflecting severe airflow limitation in Stage IV due to the advanced and homogeneous disease severity at this stage, consistent with GOLD criteria. This limited variability may reflect stringent inclusion criteria or uniform disease progression. The observed FEV1% decline correlates with functional limitations and reduced quality of life: Stage I patients experience minimal symptoms, Stage II introduces exertional dyspnea and therapy initiation, Stage III results in significant activity impairment and frequent exacerbations, and Stage IV is characterized by severe hypoxemia, reliance on oxygen therapy, and high mortality risk. Clinically, FEV1% thresholds guide therapeutic interventions such as bronchodilators (FEV1 < 80%), oxygen therapy (SpO_2_ ≤ 88%), and pulmonary rehabilitation, emphasizing the importance of timely, stage-specific management to enhance patient outcomes.

Heart rate variability (HRV)

The heart rate variability parameters, including time-domain and frequency-domain indices, showed significant differences across COPD stages, indicating progressive autonomic dysfunction with disease severity.

Time-Domain Parameters

The time-domain parameters, the standard deviation of NN intervals (SDNN) and the root mean square of successive differences (RMSSD), decreased significantly with advancing COPD severity (P < 0.001) (Table 10).

**Table 10:** Time-domain parameters P-value by ANOVA. P < 0.05 is statistically significant. COPD: chronic obstructive pulmonary disease, SDNN: standard deviation of N-N, RMSSD: root mean square of successive differences, ANOVA: analysis of variance, SD: standard deviation

COPD stage	SDNN range (ms)	Mean ± SD (ms)	RMSSD range (ms)	Mean ± SD (ms)
Stage I	50.4-84.6	64.74 ± 8.56	52.7-73	60.41 ± 5.07
Stage II	40.1-79	54.09 ± 13.82	31.4-73.9	48.29 ± 13.77
Stage III	32.5-49.9	40.67 ± 5.72	37.5-53.9	42.1 ± 3.49
Stage IV	33-43	37.12 ± 2.59	32.9-51	39.36 ± 3.8

The heart rate variability parameters, including time-domain and frequency-domain indices, showed significant differences across COPD stages, indicating progressive autonomic dysfunction with disease severity. The time-domain parameters, the standard deviation of NN intervals (SDNN) and the root mean square of successive differences (RMSSD), decreased significantly with advancing COPD severity (P < 0.001). The ranges for these parameters were particularly wide in Stages II and III, likely due to individual differences in disease progression, comorbidities, treatment adherence, and variations in autonomic reactivity. Reduced HRV, reflected in lower SDNN and RMSSD, signifies diminished parasympathetic activity and increased sympathetic dominance, correlating with heightened cardiovascular risk, including arrhythmias and mortality. Furthermore, the reductions in HRV align with other physiological changes such as declining peripheral oxygen saturation (SpO_2_) and forced expiratory volume (FEV1%), emphasizing the systemic nature of COPD. Hypoxemia likely exacerbates autonomic imbalance, contributing to the observed HRV reductions and underlining the importance of integrated cardiovascular and respiratory care in COPD management.

R-R Interval

The mean R-R interval distribution, a time-domain HRV parameter, significantly decreased with COPD severity (P < 0.001), indicating reduced cardiac autonomic regulation (Table 11).

**Table 11:** R-R interval distribution P-value by ANOVA. P < 0.05 is statistically significant. COPD: chronic obstructive pulmonary disease, ANOVA: analysis of variance, SD: standard deviation

COPD stage	R-R interval range (ms)	Mean ± SD (ms)
Stage I	736-993.9	809.15 ± 60.19
Stage II	698-936	789.46 ± 68.86
Stage III	589-745	647 ± 51.67
Stage IV	500-734	649.47 ± 52.95

The mean R-R interval was significantly reduced with advancing stages of COPD. Specifically, Stage III exhibited a mean R-R interval of 647 ms, while Stage IV showed a closely comparable value of 649.47 ms. This observation may indicate a potential plateau effect in autonomic dysfunction at the most advanced stages of COPD. Such a trend could reflect the physiological ceiling in autonomic derangements, where further progression in COPD severity does not proportionally exacerbate cardiac autonomic dysregulation. Alternatively, this convergence might also stem from limitations in sample variability, as the patient cohort for Stages III and IV may not fully capture the range of autonomic dysfunction.

A shortened R-R interval is a critical marker of autonomic imbalance, characterized by an increase in sympathetic activity and a reduction in parasympathetic tone. This imbalance disrupts normal cardiac regulation, which in turn may lead to heightened cardiovascular risk, increased symptomatology, and poorer overall prognosis in COPD patients. Specifically, reduced R-R intervals are indicative of diminished heart rate variability (HRV), a non-invasive marker of autonomic function. In COPD, this reduction exacerbates the systemic burden by promoting a cycle of hypoxemia-driven sympathetic activation, which can amplify inflammation, vascular remodeling, and right ventricular strain.

While the p-value associated with the changes in the R-R interval is statistically significant, its clinical implications warrant careful consideration. The observed differences, although meaningful from an analytical perspective, should be evaluated for their impact on clinical decision-making. For instance, the small numerical differences between Stages III and IV suggest that while autonomic dysfunction is a hallmark of advanced COPD, the incremental changes at these stages may not necessitate alterations in clinical management. However, the consistent reduction in R-R intervals across stages highlights the need for ongoing monitoring of autonomic function as part of a comprehensive COPD management strategy. This is particularly relevant for identifying high-risk patients who might benefit from targeted interventions, such as pulmonary rehabilitation or therapies aimed at modulating autonomic balance.

Frequency-domain parameters

The frequency-domain indices, including low frequency (LF), high frequency (HF), and LF/HF ratio, showed distinct trends (Table 12).

**Table 12:** Frequency-domain parameters P-value by ANOVA. P < 0.05 is statistically significant. COPD: chronic pulmonary obstructive disease, LF: low frequency, HF: high frequency, ANOVA: analysis of variance, SD: standard deviation

COPD stage	LF range	Mean ± SD	HF range	Mean ± SD	LF/HF range	Mean ± SD
Stage I	26.54-51.54	39.18 ± 8.04	46.19-73.48	59.63 ± 9.09	0.36-1.03	0.69 ± 0.23
Stage II	53.48-67.89	59.3 ± 4.9	33.8-46.51	41.06 ± 4.58	1.14-1.95	1.47 ± 0.29
Stage III	67.5-79.87	72 ± 4.27	22-32.49	28.66 ± 3.17	2.07-3.59	2.56 ± 0.47
Stage IV	67.78-90.68	79.93 ± 7.25	9.31-32.86	20.05 ± 7.25	2.06-9.73	4.72 ± 2.3

LF increased, HF decreased, and the LF/HF ratio progressively rose with COPD severity, indicating a shift toward sympathetic dominance (P < 0.001). The LF/HF ratio for Stage IV exhibited a wide range (2.06-9.73), reflecting considerable variability within this group, potentially due to outliers, sample heterogeneity, or measurement challenges. Outliers might include patients with extreme autonomic responses caused by comorbid conditions or acute physiological changes, while sample heterogeneity in demographics and clinical factors such as hypoxemia or medication use could contribute to this spread. Additionally, short-term HRV analysis may capture transient fluctuations, which might exaggerate the variability. Despite this, the overall trend of a progressively increasing LF/HF ratio from Stage I to Stage IV remained statistically significant (P < 0.001), although the variability in Stage IV warrants cautious interpretation. Overlapping LF and HF ranges between Stages III and IV (e.g., LF for Stage III: 67.5-79.87 and Stage IV: 67.78-90.68) may indicate transitional autonomic states or physiological compensation in Stage IV patients, making the boundaries between these stages less distinct. These overlaps and variabilities do not undermine the overall conclusions of increasing sympathetic dominance with disease progression but highlight the need for careful application of HRV metrics and consideration of confounding factors in clinical practice. Future research should aim to address these challenges by reducing heterogeneity and employing consistent methodologies to enhance HRV's utility as a biomarker for COPD severity.

Peripheral Oxygen Saturation (SpO_2_)

The peripheral oxygen saturation (SpO_2_) levels showed a significant decline with increasing COPD severity (P < 0.001) (Table 13).

**Table 13:** Peripheral oxygen saturation distribution P-value by ANOVA. P < 0.05 is statistically significant. COPD: chronic obstructive pulmonary disease, SpO_2_: peripheral oxygen saturation, ANOVA: analysis of variance, SD: standard deviation

COPD stage	SpO_2_ range (%)	Mean ± SD (%)
Stage I	96-99	98 ± 0.86
Stage II	94-98	95.9 ± 1.25
Stage III	92-95	94 ± 0.86
Stage IV	89-94	91.55 ± 1.39

Lower SpO_2_ values in advanced stages highlight worsening respiratory efficiency and hypoxemia. The declining SpO_2_ levels observed in advanced COPD stages not only underscore worsening hypoxemia but also highlight broader clinical implications for disease management and progression to respiratory failure. Lower oxygen saturation can exacerbate systemic effects, including reduced functional capacity, impaired quality of life, and heightened symptom burden, such as dyspnea and fatigue, which further limit daily activities and increase dependence on supplemental oxygen. While the study identifies a correlation between SpO_2_ and HRV, this relationship was qualitatively observed rather than quantitatively analyzed, leaving room for future research to explore its predictive value. The declining SpO_2_ emphasizes the need for proactive interventions, such as optimized oxygen therapy, pulmonary rehabilitation, and regular monitoring, to mitigate the risks of respiratory and cardiovascular complications, thereby improving patient outcomes in advanced COPD stages.

HRV parameters showed a marked decline as the severity of COPD increased. Specifically, time-domain measures such as SDNN and RMSSD decreased significantly, indicating a progressive reduction in overall heart rate variability and parasympathetic activity. Similarly, frequency-domain indices revealed an increase in low-frequency (LF) components and the LF/HF ratio, signifying a shift toward sympathetic dominance and heightened autonomic imbalance in advanced COPD stages. Peripheral oxygen saturation (SpO_2_) levels also declined significantly with disease severity, with Stage I patients having the highest mean SpO_2_ values (98 ± 0.86%) and Stage IV patients exhibiting the lowest levels (91.55 ± 1.39%). This progressive reduction in SpO_2_ reflects worsening pulmonary dysfunction and impaired gas exchange as COPD advances. The study found a strong correlation between reduced HRV and declining SpO_2_ levels, suggesting a close link between autonomic dysfunction and hypoxemia in COPD. Hypoxemia, a hallmark of severe COPD, triggers sympathetic activation via peripheral and central chemoreceptors, exacerbating the imbalance in autonomic regulation. This sympathetic overdrive is further amplified by systemic inflammation, another characteristic of COPD, which disrupts endothelial function and promotes oxidative stress. These mechanisms collectively impair vagal tone and enhance sympathetic activity, as evidenced by the progressive increase in the LF/HF ratio observed in this study. This intricate interplay between hypoxemia and systemic inflammation underpins the systemic nature of COPD and its associated cardiovascular risks. These findings underscore the systemic nature of COPD and its impact on both respiratory and cardiovascular regulation.

## Discussion

This study investigated the relationship between heart rate variability (HRV) and peripheral oxygen saturation (SpO_2_) levels across different stages of chronic obstructive pulmonary disease (COPD). The findings reveal significant alterations in autonomic function and oxygen saturation with increasing severity of COPD, providing insight into the systemic effects of the disease on cardiovascular and respiratory health. However, while correlations between HRV and SpO_2_ were observed, the distinction between correlation and causation must be underscored. The cross-sectional nature of this study precludes definitive causal inferences, as both HRV and SpO_2_ could be influenced by shared underlying factors, such as systemic inflammation or hypoxia-induced pathophysiological changes.

The analysis of HRV parameters demonstrated a progressive decline in both time-domain and frequency-domain indices as COPD severity increased. The standard deviation of normal-to-normal (SDNN) intervals and the root mean square of successive differences (RMSSD) showed significant reductions in advanced stages of COPD, reflecting a decrease in overall HRV and diminished parasympathetic activity. These findings align with previous studies suggesting that reduced HRV is indicative of autonomic dysfunction, which is often observed in chronic diseases such as COPD [12,13,15,16]. The decrease in RMSSD, a marker of short-term HRV influenced predominantly by parasympathetic activity, points to an impaired ability of the autonomic nervous system (ANS) to regulate heart rate effectively in response to physiological demands. This autonomic imbalance was further supported by frequency-domain analyses, where an increase in low-frequency (LF) power and the LF/HF ratio was observed, indicating heightened sympathetic activity and reduced vagal tone in severe COPD stages. Such changes in autonomic regulation may contribute to the increased cardiovascular risk associated with COPD [15,17].

SpO_2_ levels also exhibited a significant decline across the COPD stages, with patients in Stage IV showing the lowest mean SpO_2_ levels (91.55 ± 1.39%). This reduction in oxygen saturation underscores the progressive nature of gas exchange impairment in COPD. Hypoxemia, a hallmark of advanced COPD, reflects the underlying pathophysiological changes, including alveolar destruction, ventilation-perfusion mismatch, and reduced diffusing capacity of the lungs. These findings are consistent with previous studies that have documented similar reductions in SpO_2_ in severe COPD and highlight the critical role of oxygen saturation as a marker of disease severity [18].

The correlation between reduced HRV and declining SpO_2_ levels observed in this study provides further evidence of the interplay between autonomic dysfunction and hypoxemia in COPD patients. Hypoxemia is known to trigger sympathetic activation, which can exacerbate autonomic imbalance and further compromise cardiovascular health [19-21]. This vicious cycle of hypoxemia and autonomic dysfunction may partly explain the high prevalence of cardiovascular comorbidities and increased mortality risk in COPD patients. The study's findings emphasize the need for an integrated approach to managing COPD, addressing both respiratory and cardiovascular health to mitigate these risks. The observed link between hypoxemia and autonomic dysfunction is likely mediated by hypoxia-induced sympathetic activation. Prolonged hypoxemia triggers chemoreceptor stimulation, leading to increased sympathetic drive and reduced parasympathetic activity, which can exacerbate autonomic imbalance. This physiological response highlights the importance of managing hypoxia effectively to mitigate its systemic effects on the cardiovascular system [19-21].

The progressive decline in HRV and SpO_2_ with increasing COPD severity has significant clinical implications. HRV analysis, being a non-invasive and cost-effective tool, can serve as an important biomarker for early detection of autonomic dysfunction and cardiovascular risk in COPD patients [13,15]. Similarly, regular monitoring of SpO_2_ levels can help assess the severity of hypoxemia and guide oxygen therapy interventions. Early identification of autonomic and respiratory impairments can facilitate timely interventions, such as pulmonary rehabilitation, pharmacological therapy, and lifestyle modifications, potentially improving patient outcomes.

From a clinical perspective, HRV analysis could be integrated into COPD management by incorporating non-invasive monitoring tools in outpatient settings. For instance, wearable devices or portable ECG monitors with HRV analysis capabilities can provide real-time assessments of autonomic function. Similarly, continuous SpO_2_ monitoring, especially in advanced stages of COPD, could guide personalized treatment plans, such as adjusting oxygen therapy or tailoring rehabilitation programs.

Therapeutic interventions targeting autonomic balance warrant further exploration. Strategies such as vagal nerve stimulation, tailored exercise regimens, and mindfulness-based practices have shown potential in improving parasympathetic tone and reducing sympathetic overdrive. For example, supervised pulmonary rehabilitation programs incorporating aerobic exercise and resistance training have demonstrated efficacy in enhancing HRV and overall functional capacity in COPD patients.

The limitations of HRV as a biomarker must also be acknowledged. Factors such as medications (e.g., beta-blockers), acute exacerbations, and comorbidities could confound HRV measurements, necessitating careful interpretation in clinical and research settings. Similarly, challenges in SpO_2_ monitoring, such as variability due to skin pigmentation, motion artifacts, or peripheral perfusion, may affect the accuracy and reliability of measurements, particularly in low-resource settings. Addressing these challenges is critical for translating these findings into practice.

The observed autonomic imbalance in COPD patients highlights the potential role of therapeutic strategies aimed at enhancing parasympathetic activity and reducing sympathetic overdrive. However, further research is needed to explore the efficacy of such interventions in this population.

Combining HRV and SpO_2_ with other biomarkers could enhance the assessment of COPD severity. For instance, incorporating inflammatory markers (e.g., C-reactive protein and interleukin-6) or imaging modalities (e.g., CT-based emphysema quantification) could provide a more comprehensive understanding of disease progression and comorbidities.

Although the study emphasized the need for further research, specific areas warrant greater focus. Longitudinal studies examining temporal changes in HRV and SpO_2_ could elucidate the dynamics of autonomic dysfunction and hypoxemia in COPD. Evaluating the impact of targeted interventions, such as supplemental oxygen, pulmonary rehabilitation, or pharmacological therapies, on HRV and clinical outcomes would provide actionable insights for optimizing care.

It is worth noting that the proportion of patients dependent on supplemental oxygen was not explicitly documented in this study. This omission represents a potential confounder, as oxygen therapy could influence both HRV and SpO_2_ values. Future studies should stratify analyses based on oxygen dependency to better understand its effects.

Finally, the role of BMI in COPD progression merits attention. In this cohort, the absence of cachexia among Stage IV patients could reflect heterogeneity in disease presentation. Elevated BMI in severe COPD stages may be linked to fluid retention or other compensatory mechanisms, warranting further exploration. These findings highlight the complex interplay of metabolic and respiratory factors in advanced COPD.

Limitations

This study has several limitations that must be acknowledged to ensure a balanced interpretation of the findings. Firstly, the sample predominantly consisted of male participants (87.5%), limiting the generalizability of the findings. Gender differences in autonomic function and COPD progression might influence the outcomes, necessitating future studies with more balanced gender representation. Additionally, the sample was drawn from a single institution in a specific geographic region, which may restrict the applicability of the findings to broader or more diverse populations. Regional demographic and environmental factors could also influence the results.

The relatively small sample size of 80 participants reduces the statistical power and limits the ability to detect smaller but clinically significant differences. A larger, multicenter cohort is recommended for future research. Furthermore, HRV was measured over a five-minute period, capturing only short-term autonomic fluctuations. This approach does not account for long-term or circadian variations, which could provide deeper insights into autonomic dysfunction in COPD. ECG changes were not evaluated, which could have provided deeper insights into cardiac function.

Participants with common COPD comorbidities, such as hypertension, diabetes, and cardiac conditions, were excluded to reduce confounding. However, this limits the findings' applicability to real-world COPD populations, where such comorbidities are prevalent. The wide range of LF/HF ratios, particularly in Stage IV patients, suggests sample heterogeneity, potential outliers, or transient fluctuations. These variations could not be fully explored or statistically controlled in the analysis.

The higher mean BMI observed in Stage IV patients, contrary to expectations of cachexia in advanced COPD, could not be fully explained. Possible factors such as fluid retention, medication effects, or sample-specific characteristics warrant further investigation in future studies.

While individual variability in HRV parameters is acknowledged, the potential influence of statistical outliers on the findings could not be explicitly addressed. Methods such as Grubbs' test or robust statistical approaches in future studies could improve data integrity. Adjustments for multiple hypothesis testing (e.g., Bonferroni correction) were not applied. This omission might increase the risk of type I errors given the numerous comparisons made.

Finally, the study period coincided with the early phase of the COVID-19 pandemic, which could have influenced healthcare access, patient recruitment, or disease management. This context was not discussed but could have introduced unintended biases. By acknowledging these limitations, the study provides a transparent and comprehensive evaluation of its scope and constraints. Future research should aim to address these issues through larger, multicenter cohorts, longitudinal designs, and broader inclusion criteria to enhance the robustness and generalizability of the findings.

## Conclusions

The study underscores the significant impact of chronic obstructive pulmonary disease (COPD) on cardiac autonomic function and oxygen saturation, particularly in the advanced stages of the disease. Early assessment and regular monitoring of heart rate variability (HRV) and SpO_2_ are critical components of comprehensive COPD care. Portable or wearable monitors could provide real-time data on autonomic function and oxygen saturation, enabling timely identification of autonomic dysfunction or hypoxemia. For example, wearable HRV monitors could alert healthcare providers to significant deviations, allowing for early interventions and potentially mitigating disease progression.

Future research should focus on longitudinal assessments of HRV, exploring changes during acute exacerbations or in response to treatments such as pulmonary rehabilitation. These studies could clarify the relationship between autonomic dysfunction and disease progression, identifying critical intervention points. Addressing autonomic dysfunction through pharmacological treatments, lifestyle changes, and technological tools such as wearable monitors may reduce cardiovascular risks and improve outcomes. While the study's limitations, such as a single-center design and short HRV analysis duration, may restrict the generalizability of its findings, these results provide a foundation for integrating autonomic and respiratory monitoring into routine COPD care, aiming to enhance patient quality of life.

## Appendices

Study proforma

Name:

Age:

Sex:

Height:

Weight:

BMI:

History of smoking: Yes/no. If yes, duration:

History of COPD or respiratory disease other than COPD: Yes/no

History of any medication: Yes/no

Vitals

Pulse:

Blood pressure:

Respiratory rate:

SpO2:

Investigations

Spirometry:

FVC:

FEV1:

FEV1/FVC:

Heart rate variability

Mean RR (ms):

RMSSD (ms):

SDNN:

LF (nu):

HF (nu):

LF/HF ratio:
